# Entropy Generation and Heat Transfer Analysis in MHD Unsteady Rotating Flow for Aqueous Suspensions of Carbon Nanotubes with Nonlinear Thermal Radiation and Viscous Dissipation Effect

**DOI:** 10.3390/e21050492

**Published:** 2019-05-13

**Authors:** Muhammad Jawad, Zahir Shah, Aurungzeb Khan, Waris Khan, Poom Kumam, Saeed Islam

**Affiliations:** 1 Department of Mathematics, Abdul Wali Khan University, Mardan 23200, Pakistan; 2 Department of Physics, Abdul Wali Khan University, Mardan 23200, Pakistan; 3 Department of Mathematics, Kohat University of Science and Technology, Kohat 26000, Pakistan; 4 Department of Mathematics, Faculty of Science, King Mongkut’s University of Technology Thonburi (KMUTT), 126 Pracha-Uthit Road, Bang Mod, Thrung Khru, Bangkok 10140, Thailand; 5 Faculty of Science, King Mongkut’s University of Technology Thonburi (KMUTT), 126 Pracha-Uthit Road, Bang Mod, Thrung Khru, Bangkok 10140, Thailand; 6 Department of Medical Research, China Medical University Hospital, China Medical University, Taichung 40402, Taiwan

**Keywords:** Magnetohydrodynamic (MHD), rotating flow, carbon nanotubes, nonlinear thermal radiation, entropy generation, HAM

## Abstract

The impact of nonlinear thermal radiations rotating with the augmentation of heat transfer flow of time-dependent single-walled carbon nanotubes is investigated. Nanofluid flow is induced by a shrinking sheet within the rotating system. The impact of viscous dissipation is taken into account. Nanofluid flow is assumed to be electrically conducting. Similarity transformations are applied to transform PDEs (partial differential equations) into ODEs (ordinary differential equations). Transformed equations are solved by the homotopy analysis method (HAM). The radiative source term is involved in the energy equation. For entropy generation, the second law of thermodynamics is applied. The Bejan number represents the current investigation of non-dimensional entropy generation due to heat transfer and fluid friction. The results obtained indicate that the thickness of the boundary layer decreases for greater values of the rotation parameter. Moreover, the unsteadiness parameter decreases the temperature profile and increases the velocity field. Skin friction and the Nusselt number are also physically and numerically analyzed.

## 1. Introduction

Applications of nanofluids in technology and science are increasing day by day, and they play an important role in various machinery and engineering applications such as detergents, microchip technology, transferences, micromechanical systems and biomedical applications. In light of these applications, researchers have applied modern techniques and have modified the base fluids by adding ultra-fine solid particles. A fast-growing field of research is micro channel cooling, floor heating and heat renewal systems in various industries, which have been flourishing in the current era. In comparison to base liquids, nanofluids have higher single-phase thermal conductivity and heat transfer coefficients.

Khan [1] scrutinized nanofluid flow for Buongiorno’s model with the transfer of heat and mass. Mahdy et al. [2] depicted Buongiorno’s model for an unsteady nanofluid flowing in a contracting cylinder in the presence of heat transfer. The flow of nanofluid through a vertical annular pipe was deliberated by Malvandi et al. [3]. Researchers have examined nanofluid flow over a stretching sheet [4,5,6]. Non-Newtonian MHD nanofluid flow through a pipe was depicted by Ellahi [7]. Jawad et al. [8] studied the nanofluid thin film flow of Sisko fluid. Nanofluid thin film flow through a stretching sheet with heat transfer was studied by Fakour et al. [9]. The flow of nanofluids with analytical techniques was investigated by Abolbashari et al. [10]. Choi [11] was the innovator who developed the word nanofluid. Nanofluids are a mixture of metallic, nanoscale, suspended particles with a base fluid. For mass, momentum and heat transport, non-homogeneous equilibrium models in nanofluids embody four equations with two components, as expressed by Buongiorno [12]. Some interesting results pertaining to the use of nanofluids can be found in [13,14,15,16]. Recently, insufficient expressive efforts for nonlinear thermal radiations have been observed in these investigations [17]. Kumar et al. [18] scrutinized the problem of entropy generation in rotating nanofluid flow. Nadeem et al. [19] examined the issue of flow of rotating fluid, including nanoparticles of titanium and copper oxide. Mabood et al. [20] explored the flow of rotating nanofluids with the effects of radiation, magnetism, the dissipation of viscosity and a heat source. Shah et al. [21,22] investigated the flow of nanofluids within a rotating system under the influences of hall current and thermal radiation. Further theoretical investigations of nanofluids using modern applications were performed by Sheikholeslami [23,24,25]. Gireesha et al. [26] explored a single-walled nanotube in an unsteady rotating flow weight transfer. Ishaq et al. [27] examined an unsteady nanofluid thin film flow with non-dimensional entropy generation through a stretched surface.

In the study of fluid flow involving nanoparticles, nanofluids have received more attention with the arrival of nanoscience. Nanofluids are 10^9^ nm sized materials such as nanotubes, nanofibers, droplets, nanoparticles, etc. The solid phase and liquid phase create a two-phase system. Moreover, stable fluids have good writing and spreading properties on hard surfaces. Using nanofluids, the thermal conductivity of fluids can be enhanced [28,29]. Sheikholeslami et al. [30,31,32] stressed the significance of nanofluids in nanotechnology. Yadav et al. [33,34] explored MHD nanofluid flow with dissimilar phenomena such as heat transfer enhancement, stability, instability, and linear and nonlinear properties in a nanofluid model.

Recently, it has been suggested that the analysis of thin film flow has pointedly contributed in different areas, such as industry, engineering and technology, etc. The study of nanofluids was improved due to their vast applications. The latest investigations of thin film flow using different models in different geometries can be seen in [35,36,37,38,39,40,41].

Entropy is the amount of unobtainable energy in a thermodynamically closed system. The total entropy remains constant in a steady-state system. Chemical reactions, thermal resistance, joule heating, diffusion and friction concerning fluid viscosity and hard surfaces within a system are irreversible processes. Entropy production minimization is necessary for the maximal usefulness of equipment [42,43,44,45,46,47,48,49]. The entropy generated by squeezing the nanofluid flow in three-dimensions between two parallel plates is considered here. Shah et al. [50,51] proposed the heat transfer model for nanofluids. Ahmad et al. [52] deliberated squeezing the time-dependent flow of viscous nanoparticles under five dissimilar shapes. Rehman et al. [53] deliberated the flow of rotating nanofluid with entropy generation included in the thermal slip. The impacts of dissimilar constraints on liquid flow and heat transfer in a rotating fluid over a stretched disk were studied recently [54]. Alnaqi et al. [55] explored nanofluid flow with the effects of a magnetic field on the convective heat transfer rate and entropy generation through an inclined square cavity equipped with a conductor fin. Moradikazerouni et al. [56] studied CPU heat sinks in computers using a structural stability method. Hajizadeh et al. [57] investigated the thermal conductivity enhancement of nano-antifreeze containing single-walled carbon nanotubes. Vo et al. [58] deliberated γ-AlOOH nano-fluid convection performance by using various shapes of nano-additives. Alsarraf et al. [59] explored nanofluid flow with different nanoparticle shapes in a mini-channel heat exchanger using a two-phase mixture model. Moradikazerouni et al. [60] investigated the effects of five different channel forms of a micro-channel heat sink in forced convection, with application to cooling a supercomputer circuit board.

The aim of the current research is to obtain an analytical solution using the homotopy analysis method (HAM) for an unsteady, MHD, and the incompressible rotating flow of carbon nano tubes nanofluid over a shrinking surface with nonlinear thermal radiation and viscous dissipation effect. Here, we consider three types of nanofluids: CuO-water, Ag-water and Au-water, where water is used as the base fluid. The impact of the first order chemical reaction is also deliberated. The problem is formulated, solved and the corresponding results are examined in detail. Finally, the impact of the physical parameters on temperature and concentration profiles are presented and analyzed.

## 2. Mathematical Formulation of the Problem

Single-walled carbon nanotube nanofluid unsteady laminar incompressible three-dimensional rotating flow is considered over a shrinking surface. The Cartesian coordinates are chosen in the *x*, *y*, and *z* dimensions. The nanofluid rotates with an angular velocity about the *z*-axis, which is denoted by $\bar{\Omega }\left(t\right)$. The surface velocity is represented by $u_{w}\left(x,t\right)$ and given as $u_{w}\left(x,t\right)=\frac{bx}{\left(1-\delta t\right)}$ in the *x* direction, $v_{w}\;\left(x,\;t\right)$ in *y* direction and $w_{w}\left(x,t\right)$ in the z direction, and $w_{w}\left(x,t\right)$ is the wall mass flux velocity. The nanofluid flow is assumed to be thermally conductive. Radiative and viscous dissipation effects are taken into account.

Using all these assumptions, the governing equations are written as [9,25,44,45]:

(1) $$\frac{\partial u}{\partial x}+\frac{\partial v}{\partial y}+\frac{\partial w}{\partial z}=0$$

(2) $$\frac{\partial u}{\partial t}+u\frac{\partial u}{\partial x}+v\frac{\partial u}{\partial y}+w\frac{\partial u}{\partial z}+\frac{2\bar{\Omega }v}{1-\delta t}=-\frac{1}{\rho }\frac{\partial p}{\partial x}+\frac{\mu_{nf}}{\rho_{nf}}\frac{\partial^{2}u}{\partial z^{2}}-\frac{\sigma \beta_{0}^{2}}{1-\delta t}u-\frac{\mu_{nf}}{k^{*}}\frac{u}{1-\delta t}$$

(3) $$\frac{\partial v}{\partial t}+u\frac{\partial v}{\partial x}+v\frac{\partial v}{\partial y}+w\frac{\partial v}{\partial z}+\frac{2\bar{\Omega }u}{1-\delta t}=-\frac{1}{\rho }\frac{\partial p}{\partial y}+\frac{\mu_{nf}}{\rho_{nf}}\frac{\partial^{2}v}{\partial z^{2}}-\frac{\sigma \beta_{0}^{2}}{1-\delta t}v-\frac{\mu_{nf}}{k^{*}}\frac{v}{1-\delta t}$$

(4) $$\frac{\partial w}{\partial t}+u\frac{\partial w}{\partial x}+v\frac{\partial w}{\partial y}+w\frac{\partial w}{\partial z}=-\frac{1}{\rho }\frac{\partial p}{\partial z}+\frac{\mu_{nf}}{\rho_{nf}}\frac{\partial^{2}w}{\partial z^{2}}$$

(5) $$\frac{\partial T}{\partial t}+u\frac{\partial T}{\partial x}+v\frac{\partial T}{\partial y}+w\frac{\partial T}{\partial z}=\alpha_{nf}\frac{\partial^{2}T}{\partial z^{2}}+\frac{1}{{\left(\rho c_{p}\right)}_{nf}}\frac{\partial q_{r}}{\partial z}+\frac{\mu_{nf}}{{\left(\rho c_{p}\right)}_{nf}}\left[{\left(\frac{\partial u}{\partial z}\right)}^{2}+{\left(\frac{\partial v}{\partial z}\right)}^{2}+{\left(\frac{\partial w}{\partial z}\right)}^{2}\right]$$

The boundary conditions are:

(6) $$\begin{array}{l} u=u_{w}\left(x,t\right),v=0,w=0,T=T_{w}\;\;\;\;\;at\;\;\;\;\;\;z=0\\ u\to 0,v\to 0,w\to 0,T\to T_{\infty }\;\;\;\;\;\;\;\;\;\;at\;\;\;\;\;\;z\to \infty \end{array}$$

where x,y and *z* are the directions of the velocity components; $\bar{\Omega }$ denotes a constant angular velocity; $\mu_{nf}$ denotes the nanofluid dynamic viscosity; $\rho_{nf}$ denotes the nanofluid density $\alpha_{nf}$; T represents the temperature of the nanofluid; $T_{w}$ and $T_{\infty }$ are the wall and the outside surface temperatures, respectively. The radiative heat flux in Equation (5) can be shown as [9,25]:

(7) $$q_{r}=-\frac{4\sigma^{*}}{3{\left(\rho c_{p}\right)}_{nf}k^{*}}\frac{\partial T^{4}}{\partial z}=-\frac{16\sigma^{*}}{3k^{*}}T^{3}\frac{\partial T}{\partial z}$$

where the Stefan–Boltzmann constant and the mean absorption coefficient are denoted by $\sigma^{*}$ and $k^{*}$, respectively. By substituting Equation (7) into Equation (4), it can be written as [6,7,8,9,25]:

(8) $$\begin{array}{l} \frac{\partial T}{\partial t}+u\frac{\partial T}{\partial x}+v\frac{\partial T}{\partial y}+w\frac{\partial T}{\partial z}\\ =\alpha_{nf}\frac{\partial^{2}T}{\partial z^{2}}+\frac{16\sigma^{*}}{{\left(\rho c_{p}\right)}_{nf}k^{*}}\left[T^{2}\frac{\partial^{2}T}{\partial z^{2}}+3T^{2}{\left(\frac{\partial T}{\partial z}\right)}^{2}\right]+\frac{\mu_{nf}}{{\left(\rho c_{p}\right)}_{nf}}\left[{\left(\frac{\partial u}{\partial z}\right)}^{2}+{\left(\frac{\partial v}{\partial z}\right)}^{2}+{\left(\frac{\partial w}{\partial z}\right)}^{2}\right] \end{array}$$

Other parameters with nanoparticle volume fraction are mathematically presented as [6,7,22,23]:

(9) $$\begin{array}{l} \rho_{nf}=\left(1-\phi +\phi \left(\frac{{\left(\rho_{s}\right)}_{CNT}}{\rho_{f}}\right)\right),\mu_{nf}=\frac{\mu_{f}}{{\left(1-\phi \right)}^{2.5}},\alpha_{nf}\\ =\left(1-\phi \right){\left(\rho_{f}\right)}_{f}+\phi {\left(\rho_{s}\right)}_{CNT},\\ {\left(\rho C_{p}\right)}_{nf}=\left(1-\phi \right){\left(\rho C_{p}\right)}_{f}+\phi {\left(\rho C_{p}\right)}_{CNT},\frac{k_{nf}}{k_{f}}\\ =\frac{1-\phi +2\phi \left(\frac{k_{CNT}}{k_{CNT}-k_{f}}\right)\ln\left(\frac{k_{CNT}+k_{f}}{2k_{f}}\right)}{1-\phi +2\phi \left(\frac{k_{f}}{k_{CNT}-k_{f}}\right)\ln\left(\frac{k_{CNT}+k_{f}}{2k_{f}}\right)}. \end{array}$$

In Equation (9), for the base fluid the volumetric heat capacity is denoted as ${\left(\rho C_{p}\right)}_{f}$ and for CNTs as ${\left(\rho C_{p}\right)}_{CNT}$. The thermal conductivity of the nanofluid, base fluid, and CNTs are denoted as $k_{nf}$, $k_{f}$ and $k_{CNT}$, respectively. The nanoparticle volume fraction is denoted by ϕ; the density viscosity of CNTs and the base fluid are represented by $\rho_{CNT}$ and $\rho_{f}$, respectively.

Similarity transformations [26] are introduced as:

(10) $$\begin{array}{l} u=\frac{bx}{\left(1-\delta t\right)}{ƒ}^{\prime }\left(\eta \right),\;v=\frac{bx}{\left(1-\delta t\right)}g\left(\eta \right),w=-\sqrt{\frac{b\nu }{\left(1-\alpha t\right)}}f\left(\eta \right),\;\eta =\sqrt{\frac{b}{\nu \left(1-\alpha t\right)}}z,\\ T=T_{\infty }\left(1+\left(1-\delta t\right)\theta \left(\eta \right)\right). \end{array}$$

Using Equation (10) and Equations (2)–(6), we obtain:

(11) $$\begin{array}{l} \frac{1}{{\left(1-\phi \right)}^{2.5}\left(\left(1-\phi \right)+\phi \frac{{\left(\rho_{s}\right)}_{CNT}}{\rho_{f}}\right)}f^{iv}-\frac{\beta^{2}}{\lambda }f^{\prime \prime }-\frac{\mu_{nf}}{{\left(1-\phi \right)}^{2.5}k^{*}b}f^{\prime \prime }\\ -\left[\lambda \left(f^{\prime }+\frac{\eta }{2}f^{\prime \prime }\right)+f^{\prime }f^{\prime \prime }-ff^{\prime \prime }-2\frac{\Omega }{b}g^{\prime }\right]=0 \end{array}$$

(12) $$\begin{array}{l} \frac{1}{{\left(1-\phi \right)}^{2.5}\left(\left(1-\phi \right)+\phi \frac{{\left(\rho_{s}\right)}_{CNT}}{\rho_{f}}\right)}g^{\prime \prime \prime }-\frac{\beta^{2}}{\lambda }g^{\prime }+\frac{\mu_{nf}}{{\left(1-\phi \right)}^{2.5}k^{*}b}g^{\prime }\\ -\left[\lambda \left(g^{\prime }+\frac{\eta }{2}g^{\prime \prime }\right)+gf^{\prime \prime }-fg^{\prime \prime }-2\frac{\Omega }{b}f^{\prime \prime }\right]=0 \end{array}$$

(13) $$\begin{array}{l} \theta^{\prime \prime }+R\left[{\left(1+\left(\theta_{w}-1\right)\theta \right)}^{3}\theta^{\prime \prime }+3{\left(1+\left(\theta_{w}-1\right)\theta \right)}^{2}\left(\theta_{w}-1\right){\theta^{\prime }}^{2}\right]+\frac{Ec}{\mathrm{Pr}}\left({f^{\prime \prime }}^{2}+{g^{\prime }}^{2}\right)\\ -\frac{1}{\mathrm{Pr}}\left[\lambda \frac{\eta }{2}\theta^{\prime }-f\theta^{\prime }\right]=0 \end{array}$$

The transformed boundary conditions are written as:

(14) $$\begin{array}{l} f\left(0\right)=0,f^{\prime }\left(0\right)=1,g\left(0\right)=0,\theta \left(0\right)=1\;\;\;\;\;\;\;\;\;\;\;\;\;\;at\;\;\;\;\;\;\;\eta =0\\ f^{\prime }\left(\eta \right)\to 0,g\left(\eta \right)\to 0,f\left(\eta \right)\to 0,\theta \left(\eta \right)\to 0\;\;at\;\;\;\;\;\eta \to \infty . \end{array}$$

where $\Omega =\frac{\omega }{b}$ is the rotation parameter, $\lambda =\frac{\delta }{b}$ is the unsteadiness parameter and $R=\frac{16\sigma^{*}T_{\infty }^{3}}{3k_{nf}k^{*}}$ is the radiation parameter. The Prandtl number is denoted by $\mathrm{Pr}=\frac{\alpha_{nf}}{\nu_{nf}}$, the Eckert number is denoted by $\mathrm{Ec}=\frac{u_{w}^{2}}{c_{p}\left(T-T_{\infty }\right)}$ and the temperature ratio parameter is denoted by $\theta_{w}=\frac{T_{w}}{T_{\alpha }}$.

### 2.1. Physical Quantities of Interest

Skin friction in the x and y directions is denoted as $C_{fx}$ and $C_{fy}$, respectively, and the Nusselt number is $Nu_{x}$. These are defined as:

(15) $$C_{fx}=\frac{\tau_{wx}}{\rho_{f}u_{w}^{2}(x,t)},C_{fy}=\frac{\tau_{wy}}{\rho_{f}u_{w}^{2}(x,t)},Nu_{x}=\frac{xq_{w}}{\left(T_{w}-T_{\infty }\right)},$$

where $\tau_{wx}$ and $\tau_{wy}$ are the surface shear stress in the x and y directions, respectively; $q_{w}$ is the surface heat flux. These can be defined as:

(16) $$\tau_{wx}=\mu_{nf}{\left(\frac{\partial u}{\partial z}\right)}_{z=0},\;\tau_{wy}=\mu_{nf}{\left(\frac{\partial v}{\partial z}\right)}_{z=0},q_{w}=-k_{nf}\left(\frac{\partial T}{\partial z}\right)+{\left(q_{r}\right)}_{z=0}.$$

Using Equations (16) and (17), we obtain:

(17) RexCfx=1(1−ϕ)2.5f″(0),RexCfy=1(1−ϕ)2.5g′(0), NuxRex=knfkf(−[1+Rθw3]θ′(0)).

The local Reynolds number is denoted by Rex=uwx/v.

### 2.2. Entropy Generation and Bejan Number

The dimensional local entropy rate per unit volume for a nanofluid is given by [42,43,44,45,46,47,48,49,50,51,52,53]:

(18) $$S_{g,t}=\frac{K_{nf}}{T_{\alpha }^{2}}\left[{\left(\nabla T\right)}^{2}+\frac{16\sigma_{e}T_{\alpha }^{3}}{3\beta_{R}K_{f}}{\left(\nabla T\right)}^{2}\right]+\frac{\mu_{nf}}{T_{\alpha }}\Phi ,$$

where $\nabla T=\frac{\partial T}{\partial x}+\frac{\partial T}{\partial y}+\frac{\partial T}{\partial z}$ and Φ represent viscous dissipation.

In this instance,

(19) $$S_{g,t}=\frac{K_{nf}}{T_{\alpha }^{2}}\left[1+\frac{16\sigma_{e}T_{\alpha }^{3}}{3\beta_{R}K_{f}}\right]{\left(\frac{\partial T}{\partial z}\right)}^{2}+\frac{\mu_{nf}}{T_{\alpha }}\left[{\left(\frac{\partial u}{\partial z}\right)}^{2}+{\left(\frac{\partial v}{\partial z}\right)}^{2}\right]$$

(20) $$S_{g,t}=S_{h}+S_{R}+S_{f}.$$

We have three sub-generators of entropy, as deduced from Equation (20). The heat transfer dimensional entropy generator is represented by $S_{h}$. Due to thermal radiation, the dimensional entropy generator is represented by $S_{R}$ and the inter-friction of the fluid layers is represented by $S_{f}$. $S_{g,c}$ is defined as:

(21) $$S_{g,c}=\frac{K_{nf}{\left(\Delta T\right)}^{2}}{L^{2}T_{\alpha }^{2}}.$$

Now, the non-dimensional Ns (Nusselt number) is defined as:

(22) $$Ns=\frac{S_{g,t}}{S_{g,c}}.$$

To evaluate the non-dimensional Ns we use Equations (19) and (21) combined with Equations (10) and (22) to obtain:

(23) Ns=Sg,tSg,c=ReL[1+43R](θ′(η))2+ReLβr(1−ϕ)2.5Ω[(f″(η))2+(g′(η))2].

Here $A=\frac{k_{nf}}{k_{f}},$ Br and ReL are the Brinkman and Reynolds numbers, respectively, and Ω is the non-dimensional temperature, which can be shown as:

(24) $$T_{c}=\frac{T_{w}-T_{\alpha }}{T_{\alpha }}$$

Equation (23) can be rewritten as:

(25) $$Ns=N_{h},+N_{R}+N_{f}$$

where the fluid friction, thermal radiation and heat transfer non-dimensional entropy generators are denoted by $N_{h}$, $N_{R}$ and $N_{f}$, respectively.

The mathematical description of the Bejan number (Be) is:

(26) $$Be=\frac{N_{h}}{N_{h}+N_{R}+N_{f}}$$

(27) Be=KnfΩ2L2Relθ′2/ReL[1+43R]θ′2+βr(1−ϕ)2.5ΩReL[(f″2)+(g′2)].

From Equation (26), it is clear that the Bejan number is limited to the unit interval [0, 1].

## 3. Solution Procedure

The modeled Equations (11)–(13) with boundary conditions from Equation (14), together with the conditions from Equations (23) and (27), are solved with HAM. The homotopy analysis method is applied due to its outstanding results in boundary layer equations. Several researchers [46,47,48,49,50] have used HAM due to it fast convergence. The preliminary guesses are selected as follows:

$L_{\hat{f}},\;L_{\hat{g}}{\;\mathrm{and}\;L}_{\hat{\theta }}$ are linear operators which are represented as.

(28) $${ϒ}_{f}(f)=\frac{d^{3}f}{df^{3}},{ϒ}_{g}(g)=\frac{d^{2}g}{dg^{2}},{ϒ}_{\theta }(\theta )=\frac{d^{2}g}{dg^{2}}{\hat{\theta }}^{\prime \prime }$$

They have the following applicability:

(29) $$\begin{array}{l} L_{f}({ϒ}_{1}+{ϒ}_{2}\eta +{ϒ}_{3}\eta^{2}+{ϒ}_{4}\eta^{3})=0,L_{g}({ϒ}_{5}+{ϒ}_{6}\eta +{ϒ}_{7}\eta^{3})=0\\ L_{\theta }({ϒ}_{8}+{ϒ}_{9}\eta )=0 \end{array}$$

where ${ϒ}_{k}(k=1,2,3,\ldots ,9)$ is constant.

## 4. Results and Discussion

**Figure 1:**
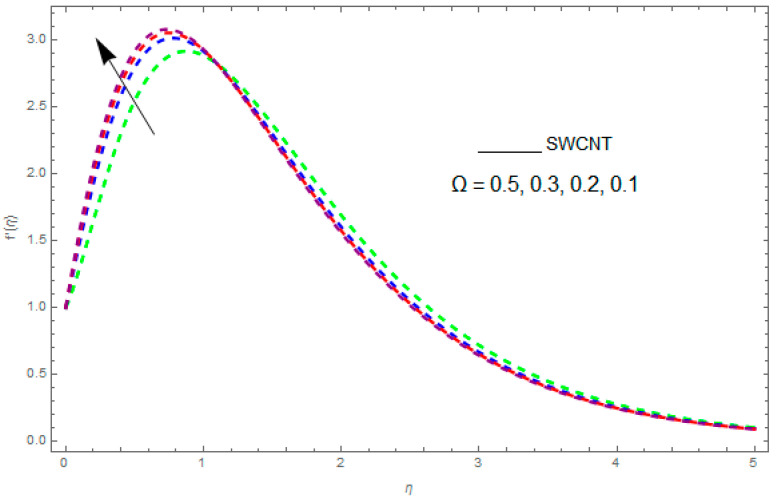
The impact of Ω on $f^{\prime }(\eta )$ when β=0.2,ϕ=0.1,λ=0.3.

**Figure 2:**
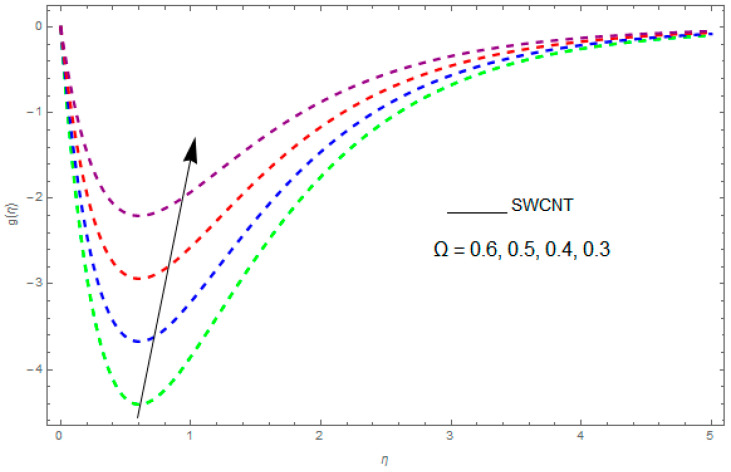
The impact of Ω on g(η) when β=0.2,ϕ=0.1,λ=0.7.

**Figure 3:**
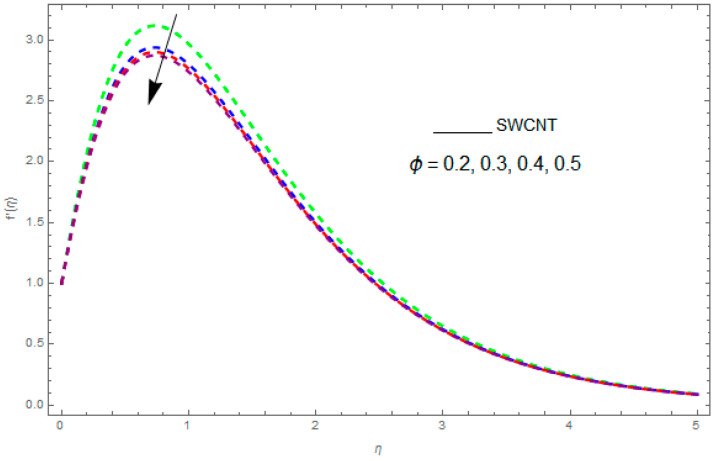
The impact of ϕ on $f^{\prime }(\eta )$ when β=0.9,λ=0.5,Ω=0.1.

**Figure 4:**
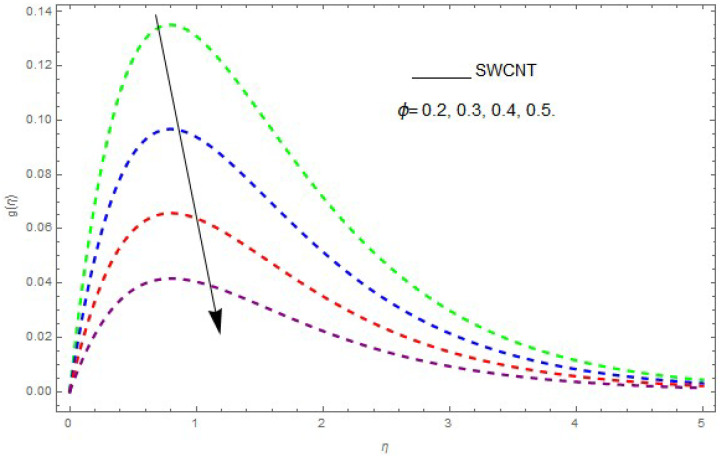
The effect of ϕ on g(η) when β=0.1,λ=0.2,Ω=0.7.

**Figure 5:**
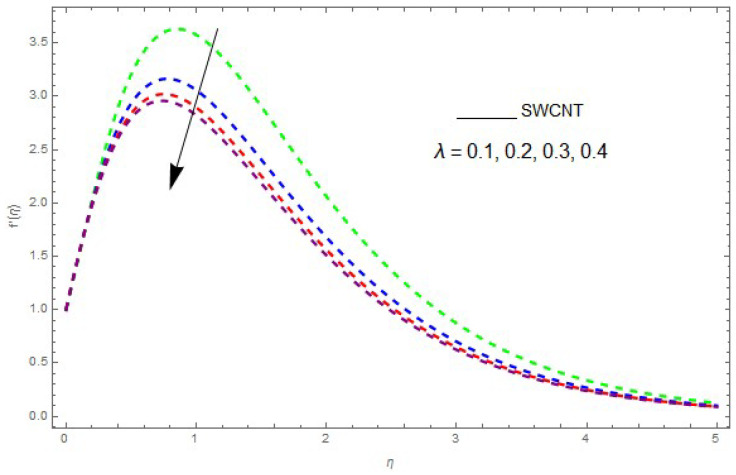
The effect of λ on $f^{\prime }(\eta )$ when β=0.9,ϕ=0.1,Ω=0.1.

**Figure 6:**
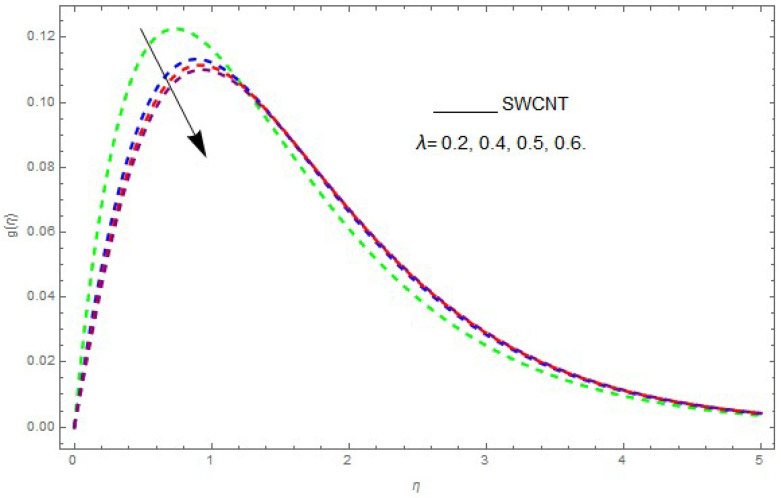
The effect of λ on g(η) when β=0.2,ϕ=0.1,Ω=0.2.

**Figure 7:**
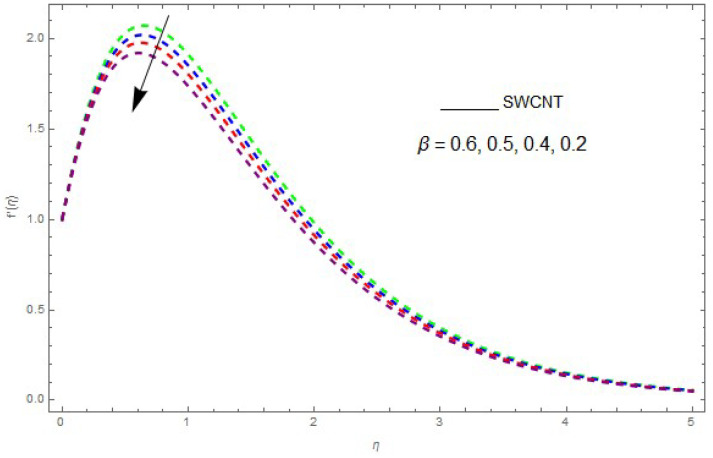
The influence of β on $f^{\prime }(\eta )$ when λ=0.2,ϕ=0.1,Ω=0.1.

**Figure 8:**
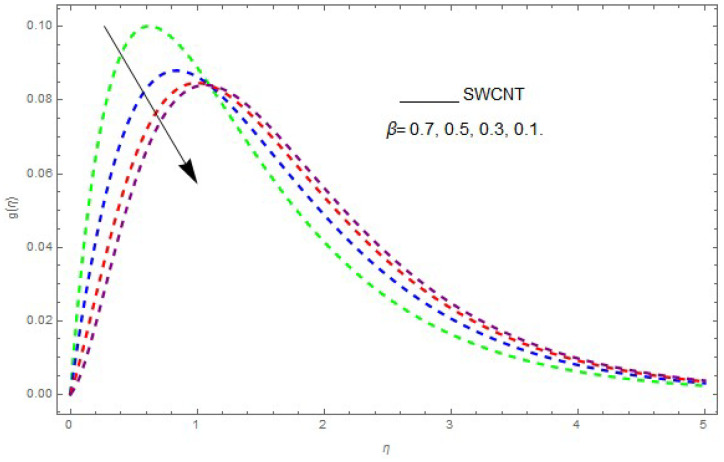
The effect of β on g(η) when λ=0.7, ϕ=0.1, Ω=0.1.

In this section, the physical outcome of dissimilar parameters of the modeled problems and their effects on $f^{\prime }(\eta )$, g(η) and θ(η) are discussed in detail. The effect of Ω, β, ϕ and λ on the velocity profile is shown in Figure 1, Figure 2, Figure 3, Figure 4, Figure 5, Figure 6, Figure 7 and Figure 8. The impact of Ω on $f^{\prime }(\eta )$ and g(η) is presented in Figure 1 and Figure 2. It can be seen that for larger values of Ω the velocity profile ($f^{\prime }(\eta )$) is increased while g(η) is decreased. Actually, increasing the rotation parameter enhances the kinetic energy, which consequently increases the velocity profile, whereas the transverse velocity (g(η)) is reduced with higher values of the rotation parameter. Figure 3 and Figure 4 represent the influence of ϕ on $f^{\prime }\left(\eta \right)$ and g(η). The higher values of ϕ reduce the velocity profiles. This is because the increase in ϕ further increases the density of the nanofluid, and as a result slows down the fluid velocity profile. Figure 5 and Figure 6 describe the effect of λ on $f^{\prime }(\eta )$ and g(η). It was perceived that increases in λ reduce the velocity profile. It is also indicated from the figure that the velocity intensifies with increasing λ, whereas we observed the opposite influence of λ on the fluid velocity inside the nanofluid and the thickness of the layer. Figure 7 and Figure 8 show the influence of β on $f^{\prime }(\eta )$ and g(η). With an increase in β the velocity profile of the fluid film is decreased. It was also detected that an increase in β results in a decrease in the fluid velocity of the nanofluid and the layer thickness. The purpose behind this influence of β by the stimulation of a lingering body force, stated as the Lorentz force, is due to the existence of β in an electrically conducting nanofluid layer. The action of this force is perpendicular to both fields. Since β represents the ratio of the viscous force to the hydromagnetic body force, a larger value of β specifies a higher hydromagnetic body force, due to which the fluid flow is reduced. The Lorentz force theory states that β has a converse consequence on $f^{\prime }(\eta )$ and g(η). Therefore, the greater values of β reduce $f^{\prime }(\eta )$ and g(η).

**Figure 9:**
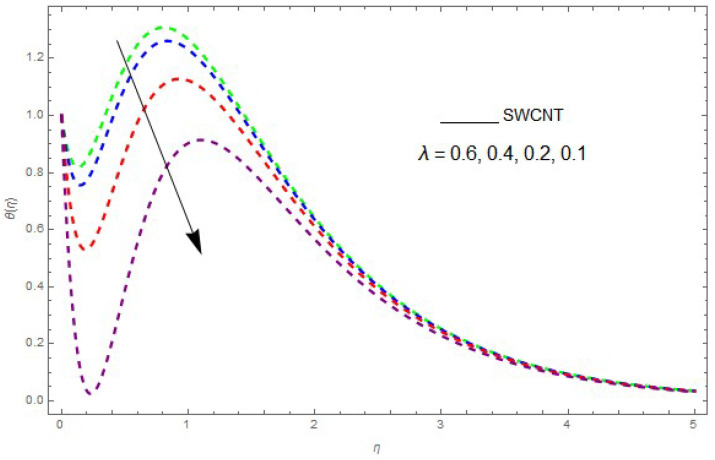
The effect of λ on θ(η) when $Rd=0.1,\;Ec=0.2,\;\mathrm{Pr}=6.2,\;\theta_{w}=1.2$.

**Figure 10:**
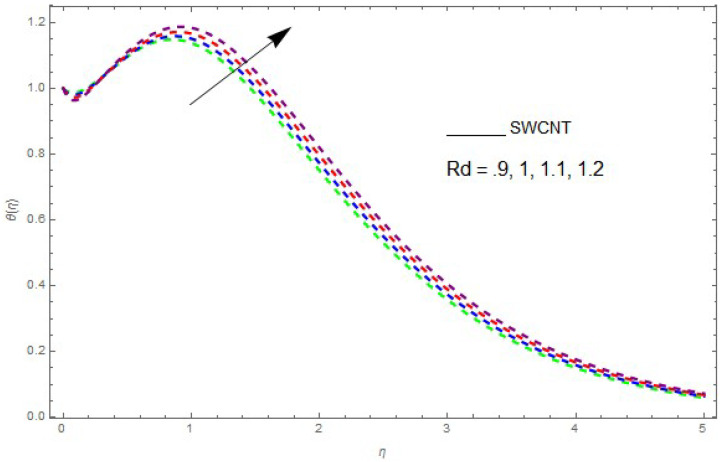
The effect of Rd on θ(η) when $Ec=0.2,\;\lambda =0.6,\;\mathrm{Pr}=6.2,\;\theta_{w}=1.2$.

**Figure 11:**
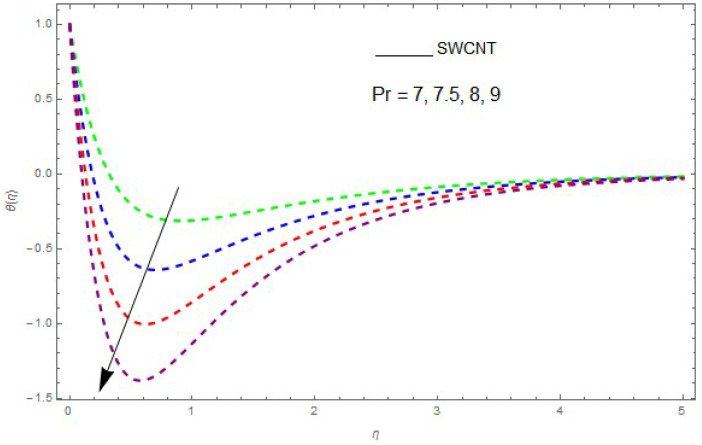
The effect of Pr on θ(η) when $Rd=0.5,\;\lambda =0.6,\;Ec=0.2,\;\theta_{w}=1.2$.

**Figure 12:**
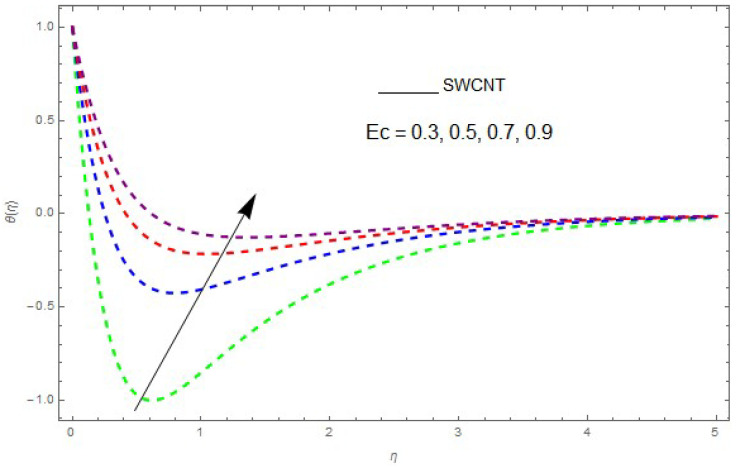
The effect of Ec on θ(η) when $Rd=0.5,\;\lambda =0.6,\;\mathrm{Pr}=6.2,\;\theta_{w}=1.2$.

**Figure 13:**
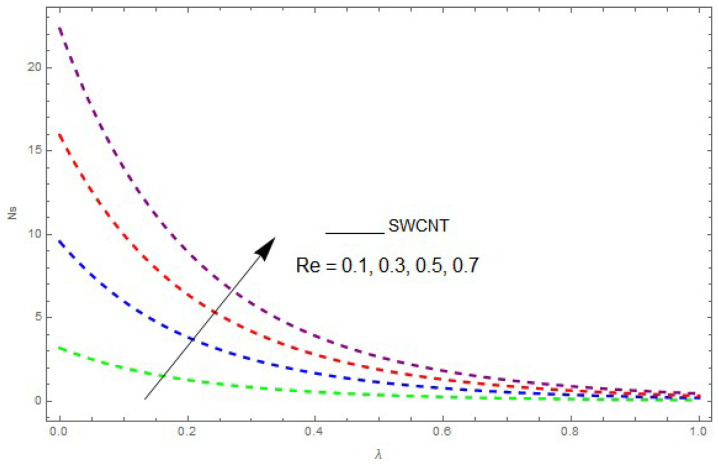
The impact of Re on entropy generation (Ns) when Ω=0.1, Br=0.2, Rd=0.1.

The influence of the physical parameters λ, Ec, Rd and Pr on θ(η) is shown in Figure 9, Figure 10, Figure 11, Figure 12 and Figure 13. Figure 9 presents the impact of λ on the θ(η) profile. Figure 9 shows that a decrease in λ reduces the boundary layer thickness. It can be seen that when unsteadiness in the stretching increases, the thin film fluid temperature and the free surface temperature are consequently reduced. Under consitions of stirring, it was revealed that greater values of λ cause the fluid temperature to fall radically, while the thickness of the thermal boundary layer is increased. The influence of Rd on θ(η) is shown in Figure 10. By increasing Rd, the temperature of the nanofluid boundary layer area increased. In fact, when Rd is raised, then it is obvious that it increases θ(η) in the boundary layer area in the fluid layer. It is shown in Figure 11 that θ(η) increases with a rise in Rd. Thermal radiation has a dominating role in the comprehensive surface heat diffusion when the coefficient of convection heat transmission is small. Increasing Rd then increases the temperature in the boundary layer area in the fluid layer. This increase leads to a drop in the rate of cooling for nanofluid flow. Therefore, the fluid θ(η) is increased. The graphical representation shows that θ(η) is increased when we increase the ratio strength and thermal radiation temperature. Thermal radiation has an important role in heat conduction when the coefficient of convection heat transmission is small. The impact of Pr on θ(η) given in Figure 11. It was observed that θ(η) decreases with larger values of Pr, while it rises for smaller values. The variation of θ(η) with respect to the variation of Pr is illustrated and shows that Pr specifies the ratio of momentum diffusivity to thermal diffusivity. It can be concluded that θ(η) decreases with increasing Pr. The nanofluids have a greater thermal diffusivity with a small Pr, but this influence does not hold for larger values of Pr; hence, θ(η) of a fluid displays a reducing behavior. Actually, the fluids having a smaller Pr have a greater thermal diffusivity, and this impact is the reverse for greater values of Pr. Based on this, a very large value of Pr causes the thermal boundary layer to drop. Figure 13 shows that with increasing Ec, θ(η) is enlarged, which is supported by the physics. By increasing Ec, heat stored in the liquid is dissipated, causing the temperature to be enhanced. θ(η) is increased with greater values of Ec and the thermal boundary layer thickness of the nanofluid becomes larger.

**Figure 14:**
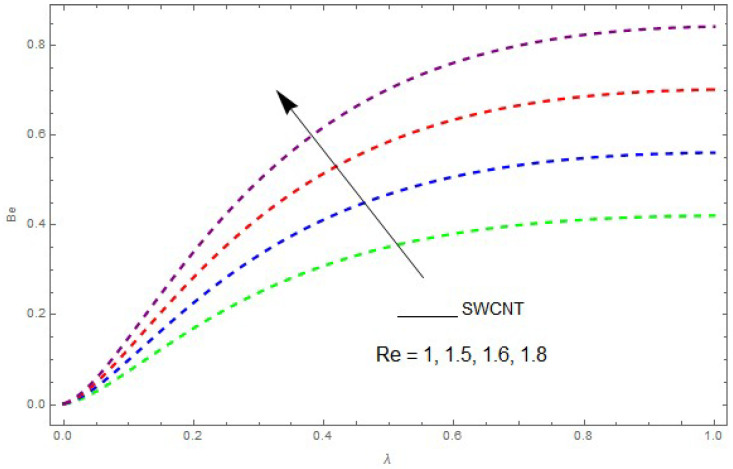
The impact of Re on the Bejan number (Be) when $Br=0.5,\;\Omega =0.5,\;Rd=0.4,\;L=1,k_{nf}=0.3$.

**Figure 15:**
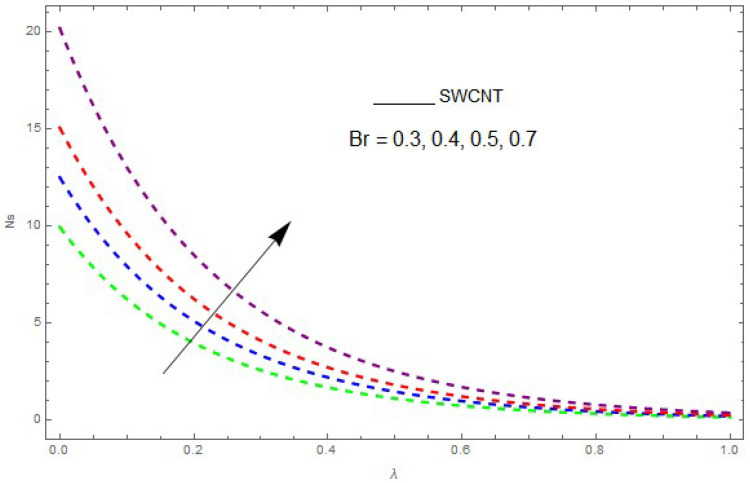
The impact of Br on entropy generation (Ns) when Ω=0.1, Re=0.2, Rd=0.1.

**Figure 16:**
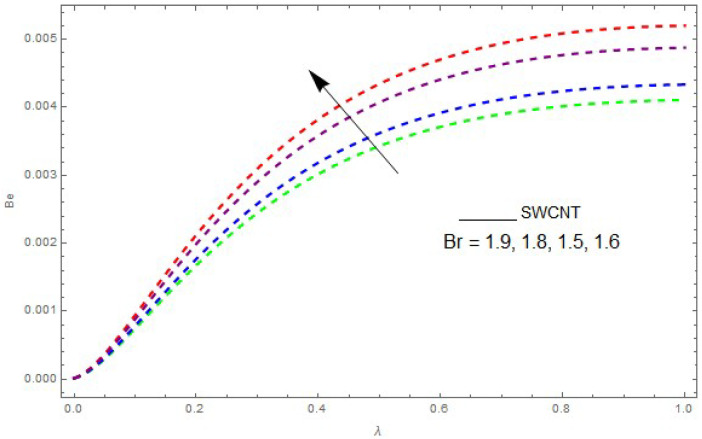
The impact of Br on the Bejan number (Be) when Re=1, Ω=0.1,Rd=0.4,L=0.9, knf=0.5.

**Figure 17:**
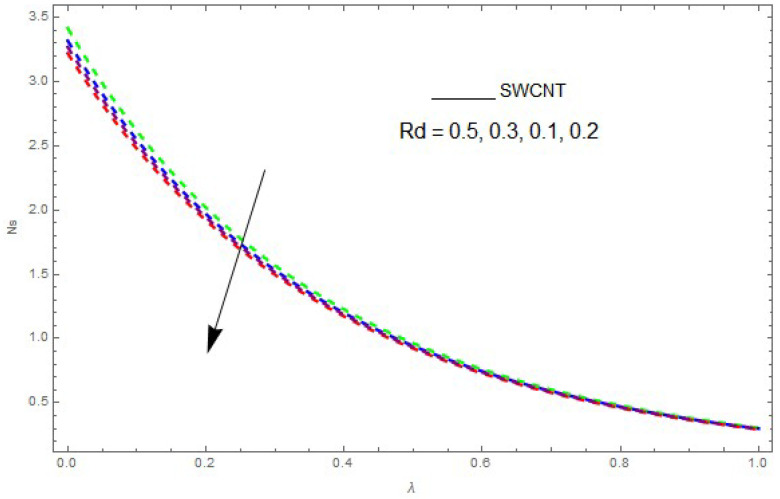
The impact of Rd on entropy generation (Ns) when Ω=0.1,Re=0.2,Br=0.3.

**Figure 18:**
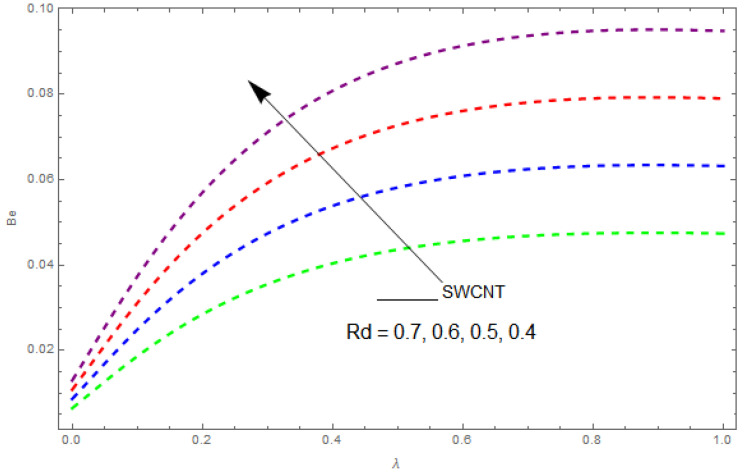
The impact of Rd on the Bejan number (Be) when Re=0.9, Ω=0.3, Br=2.5, L=0.3, knf=0.3.

**Figure 19:**
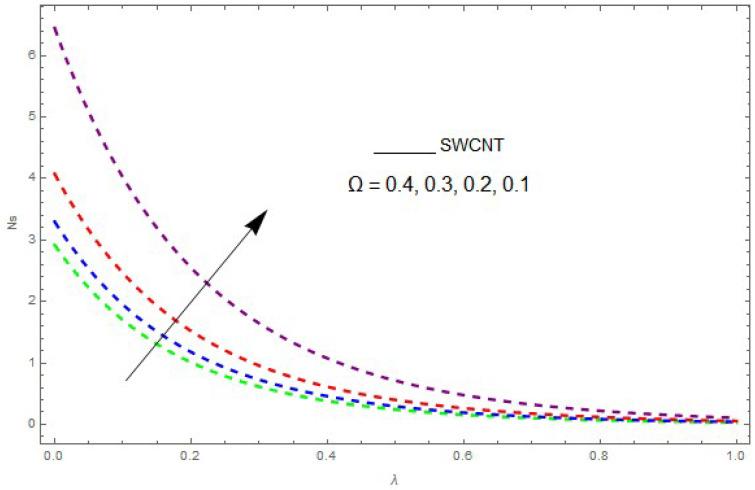
The impact of Ω on entropy generation (Ns) when Rd=0.1, Re=0.2, Br=0.5.

**Figure 20:**
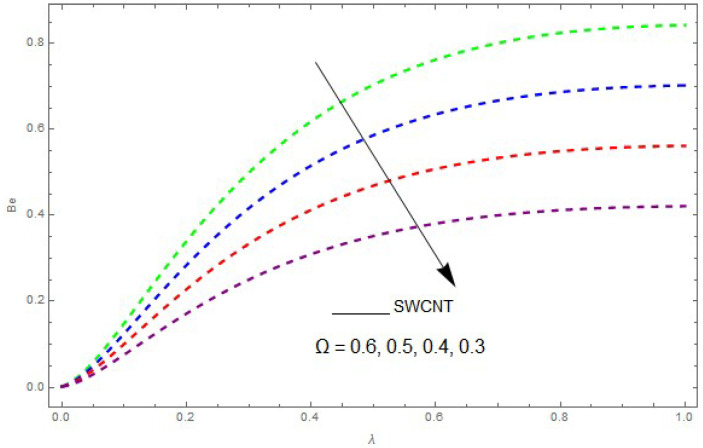
The impact of Ω on the Bejan number (Be) when Re=1, Rd=0.4, Br=0.5, L=0.3, knf=0.3.

Now, we analyze the impact of the parameters that perform a role in entropy generation and the Bejan number (Equations (23) and (27)). The influence of Re, Br, Rd and Ω on Ns and Be are examined and displayed in Figure 14, Figure 15, Figure 16, Figure 17, Figure 18, Figure 19 and Figure 20. Figure 14, Figure 15, Figure 16 and Figure 17 examine one of the significant features of this study, i.e., volumetric entropy generation for Br and Re. The influence of Ns becomes increasingly important to all these parameters. Higher Ns and Be values are due to an increase of *Br*. The higher Ns and Be values are also generated by the role of Re. Ns and Be strongly depend on Re. We observed that increasing Re also increases Ns. As Re increases, hectic motion occurs, the fluid moves more vigorously and thus the impact of heat transfer and fluid friction on Ns and Be tends to increase entropy generation. Figure 18 shows that entropy generation is reduced with increased Rd. From Figure 20, it can be seen that Be increases with an increase in Rd. From Figure 19 and Figure 20, it can be observed that Be is reduced near the lower plate of the channel where Ω is more intense; meanwhile, farther from the plate the drift is reversed due to further contribution from the irreversible heat transfer on Ns and Be, which reduces the nearby upper plate of the channel with an increase in Ω.

**Figure 21:**
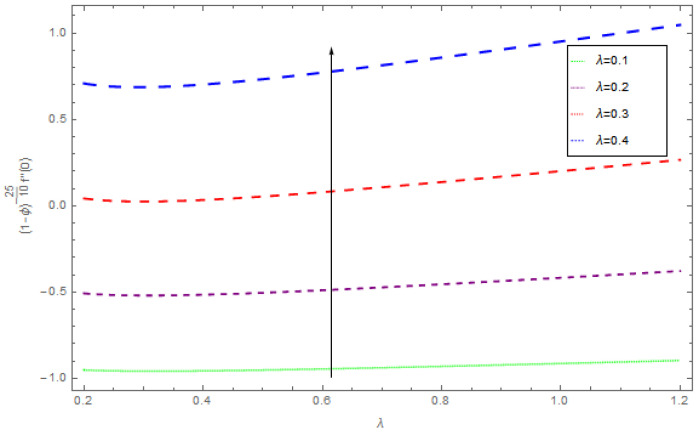
The impact of λ on skin friction when $\beta =0.3,\;\rho_{nf}=0.5,\;\Omega =0.4,\;\phi =0.1$.

**Figure 22:**
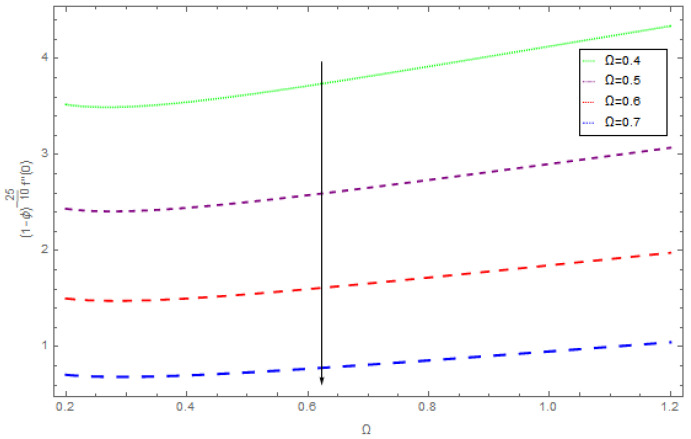
The impact of Ω on skin friction when $\beta =0.4,\;\rho_{nf}=0.2,\;\phi =0.5,\;\Omega =0.3$.

**Figure 23:**
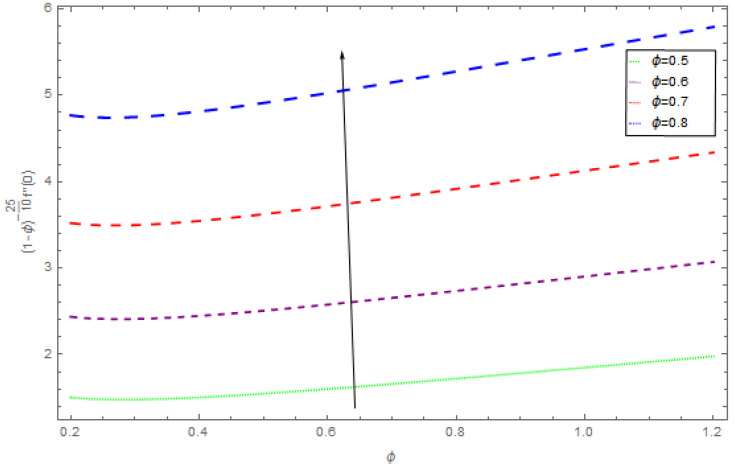
The impact of ϕ on skin friction when $\beta =0.5,\;\rho_{nf}=0.5,\;\lambda =0.5,\;\Omega =0.4$.

**Figure 24:**
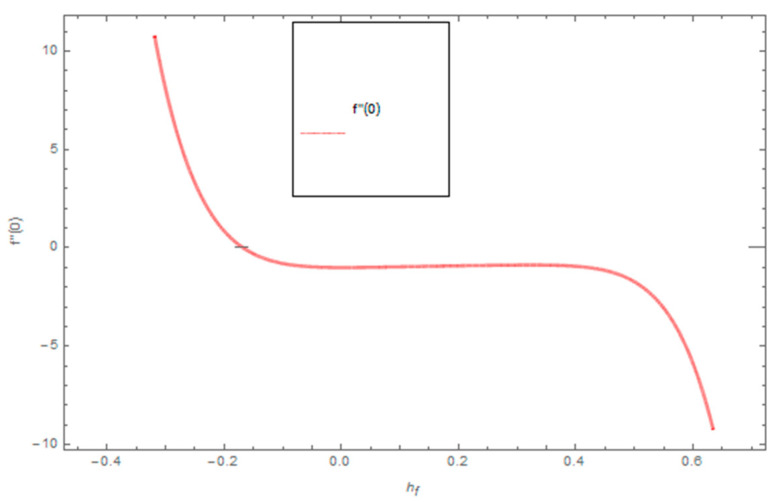
The combined graph of h-curve for $f^{\prime \prime }(0)$.

**Figure 25:**
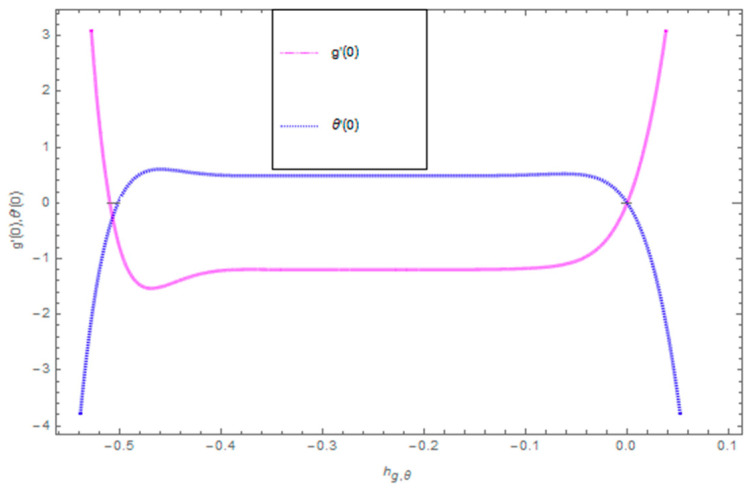
The combined graph of h-curves for $g^{\prime }(0),\theta^{\prime }(0)$.

Figure 21, Figure 22 and Figure 23 show the influence of Ω,λ and ϕ on $C_{fx}$ and $C_{fy}$. From Figure 21 it can be observed that the unsteady parameter increases $C_{fx}$ and $C_{fy}$. However, this trend is reversed for greater values of Ω. It can be seen in Figure 22 that the skin friction coefficient reduces for increasing values of Ω. Figure 23 shows that for increasing values of ϕ, the skin friction coefficient increases. From the convergence of the series given in Equation (25), f(η), g(η),θ(η) depends entirely upon the auxiliary parameters $\hbar_{f},\;\hbar_{g},\;\hbar_{\theta }$ and the so-called ℏ-curve. It is selected in such a way that it controls and converges on the series solution. The probable selection of ℏ can be found by plotting ℏ-curves of $f^{\prime \prime }(0),g^{\prime }(0),\theta^{\prime }(0)$ for the 20th order approximated HAM solution, as shown in Figure 24 and Figure 25. The valid region of ℏ is $-0.1<\hbar_{f}<0.3,-0.5<\hbar_{g}<0.1,-0.5<\hbar_{\theta }<0.1$.

## 5. Conclusions

The exploration of nanoparticles preparations has introduced more deliberation in mechanical and industrial engineering owing to their probable use for increasing the continuous phase fluid thermal performance of cooling devices. A significant source of renewable energy is thermal radiation, which can be beneficial to govern overall population levels. In the present work, the second law of thermodynamics is applied in terms of the impact of nanoparticles on non-dimensional entropy for rotating flow with suggested thermal radiation. Mathematical modeling is established by modeling five different types of nanoparticles with the purpose of achieving an appropriate mechanism to enhance the thermal conductivity of continuous phase fluid. The following conclusions can be made:

- The unsteadiness parameter decreases the temperature profile and increases the velocity field.
- The thermal boundary layer thickness is reduced for larger values of the rotation rate parameter.
- The heat transfer rate rises for greater values of Rd and $\theta_{w}$.
- With increasing values of Pr, the heat profile θ(η) reduces.
- The performance of Be is examined for the optimal values of the parameters at which Ns decreases.
- Entropy generation is increased with the increase of Pr, Ec and radiative heat flux.
- Velocity and temperature profiles decrease due to the increased unsteadiness parameter.
- Greater values of ϕ increase the frictional force within the fluid motion.

## Nomenclature

Be	Bejan number	$S_{h},S_{R},S_{f}$	Dimensional entropy generation
Br	Brinkman number	$S_{g,c}$	Characteristic entropy generation
$c_{p}$	Specific heat, J/kg·K	$T_{\infty }$	Outside surface temperature K
$C_{fx},C_{fy}$	Skin friction coefficient in *x* and *y* directions	$T_{w}$	Wall temperature K
Ec	Eckert number	T	Fluid temperature, K
h	Distance between the plates, m	$\tau_{wx},\tau_{wy}$	Surface shear stress
$k_{nf}$	Thermal conductivity of the nanofluid, W/m·K	X, Y	Topological space
Ns	Non-dimensional entropy generation	x,y,z	Coordinates
$Nu_{x}$	Nusselt number	$u_{w}$	Stretching velocity m/s
O	Origin	$v_{w}$	Surface velocity, m/s
Pr	Prandtl number	$w_{w}\left(x,t\right)$	Wall mass flux velocity m/s
P	Fluid pressure, ${N/m}^{2}$	u, v, w	Velocity components, m/s
$q_{r}$	Radioactive heat flux	$\theta_{w}$	Temperature ratio parameter
$q_{w}$	Surface heat flux, ${W/m}^{2}$	φ	Viscous dissipation
$R_{e}{}_{x}$	Local Reynolds number	ϕ	Nanoparticle volume friction
R	Radiation parameter		
**Greek Letters**			
α	Stretching parameter	$\alpha_{nf}$	Thermal diffusivity, $m^{2}/s$
δ	Transpiration parameter	η	Similarity variable
Ω	Angular velocity	$k^{*}$	Mean absorption coefficient
$\mu_{nf}$	Dynamic viscosity, kg/ms	υ	Kinematic coefficient of viscosity, $m^{2}/s$
$\rho_{f}$	Base fluid density, ${\mathrm{kg}/m}^{3}$	$\rho_{CNT}$	Density viscosity of CNT, ${\mathrm{kg}/m}^{3}$
$\rho_{nf}$	Density of the nanofluid ${\mathrm{kg}/m}^{3}$	τ	Embedding parameter where 0≤τ≤1
$\sigma^{*}$	Stefan–Boltzmann constant	ℏ	Assisting parameter
λ	Unsteadiness parameter		

## References

[1] Khan W.A. (2013). Buongiorno model for nanofluid Blasius flow with surface heat and mass fluxes. J. Thermophys. Heat Transf..

[2] Mahdy A., Chamkha A. (2015). Heat transfer and fluid flow of a non-Newtonian nano fluid over an unsteady contracting cylinder employing Buongiorno’smodel. Int. J. Numer. Method Heat Fluid Flow.

[3] Malvandi A., Moshizi S.A., Soltani E.G., Ganji D.D. (2014). ModifiedBuongiorno’s model for fully developed mixed convection flow of nanofluids in a vertical annular pipe. Comput. Fluids..

[4] Hayat T., Ashraf M.B., Shehzad S.A., Abouelmaged E.I. (2015). Three dimensional flow of Erying powell nanofluid over an exponentially stretching sheet. Int. J. Numer. Method Heat Fluid Flow.

[5] Nadeem S., Haq R.U., Akbar N.S., Lee C., Khan Z.H. (2013). Numerical study of boundary layer flow and heat transfer of Oldroyed-B nanofluid towards a stretching sheet. PLoS ONE.

[6] Rosmila A.B., Kandasamy R., Muhaimin I. (2012). Lie symmetry group’s transformation for MHD natural convection flow of nanofluid over linearly porous stretching sheet in presence of thermal stratification. Appl. Math. Mech. Engl. Ed..

[7] Ellahi R. (2013). The effects of MHD and temperature dependent viscosity on the flow of non-Newtonian nanofluid in a pipe Analytical solutions. Appl. Math. Model..

[8] Jawad M., Shah Z., Islam S., Islam S., Khan W., Khan Z.A. (2019). Nanofluid thin film Flow of Sisko Fluid and Variable Heat Transfer over an Unsteady Stretching Surface with External Magnetic Field. J. Algorithms Comput. Technol..

[9] Abolbashari M.H., Freidoonimehr N., Rashidi M.M. (2015). Analytical modeling of entropy generation for Casson nano-fluid flow induced by a stretching surface. Adv. Powder Technol..

[10] Nadeem S., Haq R.U., Khan Z.H. (2014). Numerical study of MHD boundary layer flow of a Maxwell fluid past a stretching sheet in the presence of nanoparticles. J. Taiwan Inst. Chem. Eng..

[11] Choi S.U.S., Siginer D.A., Wang H.P. (1995). Enhancing thermal conductivity of fluids with nanoparticle developments and applications of non-Newtonian flows. ASME N. Y..

[12] Buongiorno J. (2005). Convective transport in nanofluids. ASME J. Heat Transf..

[13] Kumar K.G., Rudraswamy N.G., Gireesha B.J., Krishnamurthy M.R. (2017). Influence of nonlinear thermal radiation and viscous dissipation on three-dimensional flow of Jeffrey nanofluid over a stretching sheet in the presence of Joule heating. Nonlinear Eng..

[14] Kumar K.G., Ramesh G.K., Gireesha B.J., Gorla R.S.R. (2017). Characteristics of Joule heating and viscous dissipation on threedimensional flow of Oldroyd B nanofluid with thermal radiation. Alex Eng. J..

[15] Rudraswamy N.G., Shehzad S.A., Kumar K.G., Gireesha B.J. (2017). Numerical analysis of MHD three-dimensional Carreau nanoliquid flow over bidirectionally moving surface. J. Braz. Soc. Mech. Sci. Eng..

[16] Gireesha B.J., Kumar K.G., Ramesh G.K., Prasannakumara B.C. (2018). Nonlinear convective heat and mass transfer of Oldroyd-B nanofluid over a stretching sheet in the presence of uniform heat source/sink. Results Phys..

[17] Kumar K.G., Gireesha B.J., Manjunatha S., Rudraswamy N.G. (2017). Effect of nonlinear thermal radiation on double-diffusive mixed convection boundary layer flow of viscoelastic nanofluid over a stretching sheet. Int. J. Mech. Mater. Eng..

[18] Kumar K.G., Rashidi M.M., Abelman S., Mehr N.F. (2013). Entropy generation in steady MHD flow due to a rotating porous disk in a nanofluid. Int. J. Heat Mass Transf..

[19] Nadeem S., Rehman A.U., Mehmood R. (2016). Boundary layer flow of rotating two phase nanofluid over a stretching surface. Heat Transf. Asian Res..

[20] Mabood F., Ibrahim S.M., Khan W.A. (2016). Framing the features of Brownian motion and thermophoresis on radiative nanofluid flow past a rotating stretching sheet with magnetohydrodynamics. Results Phys..

[21] Shah Z., Islam S., Gul T., Bonyah E., Khan M.A. (2018). The electrical MHD and hall current impact on micropolar nanofluid flow between rotating parallel plates. Results Phys..

[22] Shah Z., Islam S., Ayaz H., Khan S. (2019). Radiative Heat and Mass Transfer Analysis of Micropolar Nanofluid Flow of Casson Fluid between Two Rotating Parallel Plates with Effects of Hall Current. ASME J. Heat Transf..

[23] Sheikholeslami M. (2018). CuO-water nanofluid flow due to magnetic field inside a porous media considering Brownian motion. J. Mol. Liq..

[24] Sheikholeslami M. (2018). Numerical Investigation of nanofluid free convection under the influence of electric field in a porous enclosure. J. Mol. Liq..

[25] Sheikholeslami M., Shah Z., Tassaddiq A., Shafee A., Khan I. (2019). Application of Electric Field for Augmentation of Ferrofluid Heat Transfer in an Enclosure Including Double Moving Walls. IEEE Access.

[26] Gireesha B.J., Ganesh K., Krishanamurthy M.R., Rudraswamy N.G. (2018). Enhancement of heat transfer in an unsteady rotating flow for the aqueous suspensions of single wall nanotubes under nonlinear thermal radiation: A numerical study. Colloid Polym. Sci..

[27] Ishaq M., Ali G., Shah Z., Islam S., Muhammad S. (2018). Entropy Generation on Nanofluid Thin Film Flow of Eyring–Powell Fluid with Thermal Radiation and MHD Effect on an Unsteady Porous Stretching Sheet. Entropy.

[28] Das S.K., Choi S.U., Yu W., Pradeep T. (2007). Nanofluids Science and Technology.

[29] Wong K.F.V., Leon O.D. (2010). Applications of nanofluids: Current and future. Adv. Mech. Eng..

[30] Sheikholeslami M., Shah Z., shafi A., khan I., Itili I. (2019). Uniform magnetic force impact on water based nanofluid thermal behavior in a porous enclosure with ellipse shaped obstacle. Sci. Rep..

[31] Li Z., Sheikholeslami M., Shah Z., Shafee A., Al-Qawasmi A., Tlili I. (2019). Time dependent heat transfer in a finned triplex tube during phase changing of nanoparticle enhanced PCM. Eur. Phys. J. Plus (EPJP).

[32] Sheikholeslami M., Rokni H.B. (2017). Simulation of nanofluid heat transfer in presence of magnetic field, A review. Int. J. Heat Mass Transf..

[33] Yadav D., Lee D., Cho H.H., Lee J. (2016). The onset of double-diffusive nanofluid convection in a rotating porous medium layer with thermal conductivity and viscosity variation: A revised model. J. Porous Media.

[34] Yadav D., Nam D., Lee J. (2016). The onset of transient Soret-driven MHD convection confined within a Hele-Shaw cell with nanoparticles suspension. J. Taiwan Inst. Chem. Eng..

[35] Saeed A., Shah Z., Islam S., Jawad M., Ullah A., Gul T., Kumam P. (2019). Three-Dimensional Casson Nanofluid Thin Film Flow over an Inclined Rotating Disk with the Impact of Heat Generation/Consumption and Thermal Radiation. Coatings.

[36] Shah Z., Dawar A., Kumam P., Khan W., Islam S. (2019). Impact of Nonlinear Thermal Radiation on MHD Nanofluid Thin Film Flow over a Horizontally Rotating Disk. Appl. Sci..

[37] Khan N.S., Gul T., Kumam P., Shah Z., Islam S., Khan W., Zuhra S., Sohail A. (2019). Influence of Inclined Magnetic Field on Carreau Nanoliquid Thin Film Flow and Heat Transfer with Graphene Nanoparticles. Energies.

[38] Khan N., Zuhra S., Shah Z., Bonyah E., Khan W., Islam S. (2018). Slip flow of Eyring-Powell nanoliquid film containing graphene nanoparticles. AIP Adv..

[39] Ullah A., Alzahrani E.O., Shah Z., Ayaz M., Islam S. (2019). Nanofluids Thin Film Flow of Reiner-Philippoff Fluid over an Unstable Stretching Surface with Brownian Motion and Thermophoresis Effects. Coatings.

[40] Shah Z., Bonyah E., Islam S., Khan W., Ishaq M. (2018). Radiative MHD thin film flow of Williamson fluid over an unsteady permeable stretching. Heliyon.

[41] Jawad M., Shah Z., Islam S., Islam S., Bonyah E., Khan Z.A. (2018). Darcy-Forchheimer flow of MHD nanofluid thin film flow with Joule dissipation and Navier’s partial slip. J. Phys. Commun..

[42] Adesanya S.O., Makinde O.D. (2014). Entropy generation in couple stress fluid flow through porous channel with fluid slippage. Int. J. Exergy.

[43] Kumam P., Shah Z., Dawar A., Rasheed H.U., Islam S. (2019). Entropy Generation in MHD Radiative Flow of CNTs Casson Nanofluid in Rotating Channels with Heat Source/Sink. Math. Probl. Eng..

[44] Sheremet M.A., Oztop H.F., Pop I., Hamdeh N.A. (2016). Analysis of entropy generation in natural convection of nanofluid inside a square cavity having hot solid block: Tiwari and das’ model. Entropy.

[45] Dawar A., Shah Z., Khan W., Idrees M., Islam S. (2019). Unsteady squeezing flow of MHD CNTS nanofluid in rotating channels with Entropy generation and viscous Dissipation. Adv. Mech. Eng..

[46] Khan M.I., Qayyum S., Hayat T., Khan M.I., Alsaedi A., Khan T.A. (2018). Entropy generation in radiative motion of tangent hyperbolic nanofluid in presence of activation energy and nonlinear mixed convection. Phys. Lett. A.

[47] Feroz N., Shah Z., Islam S., Alzahrani E.O., Khan W. (2019). Entropy Generation of Carbon Nanotubes Flow in a Rotating Channel with Hall and Ion-Slip Effect Using Effective Thermal Conductivity Model. Entropy.

[48] Alharbi S.O., Dawar A., Shah Z., Khan W., Idrees M., Islam S., Khan I. (2018). Entropy Generation in MHD Eyring–Powell Fluid Flow over an Unsteady Oscillatory Porous Stretching Surface under the Impact of Thermal Radiation and Heat Source/Sink. Appl. Sci..

[49] Hayat T., Khan M.I., Qayyum S., Alsaedi A., Khan M.I. (2018). New thermodynamics of entropy generation minimization with nonlinear thermal radiation and nano materials. Phys. Lett. A.

[50] Shah Z., Dawar A., Islam S., Khan I., Ching D.L.C. (2018). Darcy-Forchheimer Flow of Radiative Carbon Nanotubes with Microstructure and Inertial Characteristics in the Rotating Frame. Case Stud. Therm. Eng..

[51] Shah Z., Dawar A., Islam S., Khan I., Ching D.L.C., Khan A.Z. (2018). Cattaneo-Christov model for Electrical MagnetiteMicropoler Casson Ferrofluid over a stretching/shrinking sheet using effective thermal conductivity model. Case Stud. Therm. Eng..

[52] Rehman A.U., Mehmood R., Nadeem S. (2017). Entropy analysis of radioactive rotating Nanofluid with thermal Slip. Appl. Therm. Eng..

[53] Sahoo B., Shevchuk I.V. (2019). Heat transfer due to revolving flow of Reiner-Rivlin fluid over a stretchable surface. Therm. Sci. Eng. Prog..

[54] Shevchuk I.V. (2006). Unsteady conjugate laminar heat transfer of a rotating non-uniformly heated disk. Int. J. Heat Mass Transf..

[55] Alnaqi A.A., Aghakhani S., Pordanjani H., Bakhtiari R., Asadi A. (2019). Effects of magnetic field on the convective heat transfer rate and entropy generation of a nanofluid in an inclined square cavity equipped with a conductor fin: Considering the radiation effect. Int. J. Heat Mass Transf..

[56] Moradikazerouni A., Afrand M., Alsarraf J., Wongwises S., Asadi A., Nguyen T.K. (2019). Investigation of a computer CPU heat sink under laminar forced convection using a structural stability method. Int. J. Heat Mass Transf..

[57] Hajizadeh A., Safaei M.R., Afrand M., Yarmand H., Zulkifli N.W.B.M. (2019). Assessment of thermal conductivity enhancement of nano-antifreeze containing single-walled carbon nanotubes: Optimal artificial neural network and curve-fitting. Phys. A Stat. Mech. Its Appl..

[58] Vo D.D., Alsarraf J., Moradikazerouni A., Afrand M., Salehipour H., Qi C. (2019). Numerical investigation of γ-AlOOH nano-fluid convection performance in a wavy channel considering various shapes of nanoadditives. Powder Technol..

[59] Alsarraf J., Moradikazerouni A., Shahsavar A., Afrand M., Salehipou H., Tran D.M. (2019). Hydrothermal analysis of turbulent boehmite alumina nanofluid flow with different nanoparticle shapes in a minichannel heat exchanger using two-phase mixture model. Phys. A Stat. Mech. Its Appl..

[60] Moradikazerouni A., Afrand M., Alsarraf J., Mahian O., Wongwises S., Tran M.D. (2019). Comparison of the effect of five different entrance channel shapes of a micro-channel heat sink in forced convection with application to cooling a supercomputer circuit board. Appl. Therm. Eng..

